# The involvement of MMP-2 and MMP-9 in heart exercise-related angiogenesis

**DOI:** 10.1186/1479-5876-11-283

**Published:** 2013-11-07

**Authors:** Marianna Bellafiore, Giuseppe Battaglia, Antonino Bianco, Felicia Farina, Antonio Palma, Antonio Paoli

**Affiliations:** 1 Department of Legal, Society and Sport Sciences, University of Palermo, Via E. Duse 2, 90146 Palermo, Italy; 2 Department of Experimental Biomedicine and Clinical Neuroscience (BioNeC), University of Palermo, Via del Vespro 129, Palermo, Italy; 3 Department of Biomedical Sciences (DSB), University of Padova, Via F. Marzolo 3, Padova, Italy; 4 Facoltà di Scienze Motorie, Via Eleonora Duse, 3 (Presso Campus Lincoln), 90146 Palermo, Italy

**Keywords:** Capillary growth, Matrix metalloproteinases, Aerobic training, Myocardiocyte, Cardiac remodelling

## Abstract

**Background:**

Little is known about the involvement of matrix metalloproteinases (MMPs) in cardiac vascular remodelling induced by exercise. Our aim was to evaluate and localize MMP-2 and MMP-9’s activities in relation to capillary proliferation in mouse hearts trained for 15, 30 and 45 days.

**Methods:**

Sixty-three mice were randomly assigned to 7 groups: four control sedentary groups (C0, C15, C30 and C45) and three groups trained by an endurance protocol (T15, T30 and T45). MMP-2 and MMP-9 were examined with zymography and immunostaining analyses. Capillary proliferation was evaluated counting the number of CD31-positive cells.

**Results:**

Different activity patterns of the latent form of both MMPs were found. Pro-MMP-9 increased after 15 days of training; whereas pro-MMP-2 gradually decreased after 30 and 45 days of training below the control groups. The latter was inversely correlated with capillary growth. MMP-9 was mainly localized in myocardiocytes and less evident in capillaries. Conversely, MMP-2 was more intense in capillary endothelial cells and slightly in myocardiocytes.

**Conclusions:**

A different spatiotemporal modulation of pro-MMP-2 and pro-MMP-9 activities has been detected in the myocardium during angiogenesis related to the aerobic training. These results can be useful to draw up training protocols for improving the performance of healthy and diseased human hearts.

## Background

Cardiac angiogenesis induced by exercise is known to be a fundamental physiological response for maintaining the function of muscle adequate to increases in metabolic demand [1,2]. It is likely that haemodynamic and mechanical events associated with modifications in the blood flow, muscle contraction and oxygen levels are key signals to trigger vascular remodelling [3,4]. White et al. showed that the appearance of new capillaries in adult swine heart was most significant in the early phases of exercise training. Capillary density increased after three weeks of endurance treadmill training and decreased at 8 weeks because capillaries developed into small arterioles as shown by the same authors [5]. These results are in agreement with our previous study reporting a progressive increase in capillary-occupied area of mouse heart during six weeks of endurance training program with the peak reached at the fourth week [2]. This vascular remodelling was the main structural adaptation contributing to the hypertrophy of left ventricle observed in our study in response to aerobic training.

It has widely been accepted that the matrix metalloproteinases (MMPs) play a key role in angiogenesis, either in pathological conditions, such as tumours [6,7], heart failure [8], and in physiological processes as the ovarian cycle [9] and exercise-induced vascular remodelling [10,11].

MMPs are zinc-dependent endoproteinases able to degrade various extracellular matrix (ECM) components and are synthesized as latent pro-enzymes activated via cleavage of the regulatory peptide sequence by serine proteases, such as plasmin, and other MMPs [6]. They can contribute to the angiogenic process in different ways including endothelial cell migration through surrounding tissues by disrupting ECM barriers [12]; the release of sequestered angiogenic factors, such as fibroblast growth factors-2 (FGF-2) or vascular endothelial growth factor (VEGF); the inhibition of angiogenesis by generating anti-angiogenic fragments from ECM molecules, such as endostatin and tumstatin [13].

The most extensively investigated MMPs in angiogenesis are MMP-2 and MMP-9, collagenases able to degrade collagen type IV (the most prevalent protein in basal lamina) and specifically modulated by exercise in skeletal muscle [10,14,15].

The strong link between MMPs and exercise-related neo-capillarization was found by Haas et al. who reported that the inhibition of MMPs activity eliminated the capillary growth induced by chronic skeletal muscle stimulation [10].

Most studies investigating the regulation of MMPs by exercise were carried out in the skeletal muscle [10,11,15]; their function in cardiac angiogenesis induced by exercise has poorly been tested. In myocardium, MMPs are produced by fibroblasts, inflammatory cells and myocytes [16] and changes in their expression and activity were associated with ECM and left ventricle remodelling after myocardial infarction [8,17].

The aim of the present study was to investigate the localization and activity patterns of MMP-2 and MMP-9 in relation to capillary proliferation of hypertrophic mouse hearts following endurance training. Since the vascular changes induced by exercise are not uniform over the course of exercise training, this study allows us to associate the activity profiles of MMP-2 and MMP-9 with the time course of capillary growth induced by endurance training in the myocardium.

## Materials and methods

### Experimental design

Sixty-three male 10-week-old Swiss mice (body weight: 38.00 ± 3.8 g) were randomly assigned to 7 groups, each of these including 9 animals by an investigator. One group was sacrificed before starting the training period and corresponded to the control group (C) at point zero (C0); three groups were selected as controls sedentary for 15 (C15), 30 (C30) and 45 days (C45) and three groups were trained by an endurance protocol for 15 (T15), 30 (T30) and 45 days (T45). Mice were trained for 5 days/week on a motorized rotor 0.20 m in circumference (Rota-Rod; Ugo Basile, Biological Research Apparatus, Comerio, Varese, Italy), at progressively increasing loads [2]. Treadmill speed was the same in the first and second week (16 rotations/min respectively for 15 and 30 min per day). In the third, fourth and fifth week, mice were trained at a speed of 20 rotations/min respectively for 30, 45 and 60 min per day. The maximum rate of exercise, achieved in the sixth week, was 24 rotations/min for 60 min per day (speed which mice could achieve without tumbling). Twenty-four hours after the completion of the last exercise session, the mice were weighed, anaesthetized with 2% haloethane in O_2_ and sacrificed.

We carried out the experiments on the same heart samples used in a previous study, in which we had observed that after 30 and 45 days of endurance training the hearts showed left ventricle hypertrophy associated with a significant increase in the area occupied by capillaries [2]. The investigation conforms to the *Guide for the Care and Use of Laboratory Animals* published by the US National Institutes of Health (NIH Publication No. 85–23, revised 1996) and it has been approved by an appropriate local ethics committee (“Palermo 1” at the University Hospital “P. Giaccone” of Palermo).

### Gelatin zymography on mono-dimensional gel electrophoresis

The enzymatic activity of MMP-2 and MMP-9 present in total tissue extracts from frozen mouse left ventricle was performed with gelatine zymography analyses. Tissue samples were homogenized in a buffer containing 50 mM Tris/HCl (pH 7.5), 150 mM NaCl and 1% Nonidet P-40 at 4°C. The homogenate was incubated for 1 hour at 4°C and then centrifuged at maximum velocity in an eppendorf micro-centrifuge for 20 minutes (min) at 4°C. The supernatant was collected and the protein concentration determined using Bradford assay (Bio-Rad, Philadelphia, USA). 20 μg of protein aliquots were used for each zymographic assay and human serum (HS) as a marker. Mono-dimensional gelatine zymography was performed under non-reducing conditions on 7.5% SDS-PAGE copolymerized with 0.1% gelatine. Following the electrophoresis, the SDS was removed from the gels by several washes with 2.5% Triton-× 100 in 50 mM Tris/HCl, pH 7.5. The zymograms were subsequently developed for 18 h at 37°C in the same buffer, in which 0.15 M NaCl, 10 mM CaCl2 and 0.02% NaN3 were added. Gels were stained with Coomassie blue and unstained areas corresponding to zones of digestion were visualized after distaining with 7% methanol in 5% acetic acid. In order to estimate MMP-2 and MMP-9 levels in control and trained mouse hearts, band intensity was quantified by computer-assisted image analysis (Adobe Photoshop 6.0, Adobe System Incorporation, USA) calculating pixel number of signal per cm^2^. Every data point is representative of three independent experiments.

### Immunostaining analyses

In order to study the capillary proliferation in myocardium following aerobic training, we evaluated the expression of CD-31 protein as shown by Kobayashi et al. [18]. The expression of CD31, MMP-2 and MMP-9 was examined with immunohistochemical analyses. Hearts were fixed with formalin, embedded with paraffin and cut to obtain 5 μm sections. After incubation of sections for 10 min with 0.3% H_2_O_2_, a serum-free protein block (DAKO, Carpinteria, USA) was added for 10 min. Before adding MMP-2 and MMP-9 primary antibodies, the slides were treated with monohydrated citrate buffer (pH 6.0, 0.01 M) in a water bath for 10 min at 100°C for the antigen retrieval. Sections were then incubated with the monoclonal antibodies against MMP-2, MMP-9 (1:100; Calbiochem®, San Diego, CA, USA) and CD31 (1:20; DAKO, Carpinteria, CA, USA) for one hour at room temperature. Anti-MMP-2 and anti-MMP-9 recognize both latent and active form. Non-immune mouse serum was substituted for negative controls (NC). After incubation for 10 min with a biotinylated secondary antibody, AEC chromogen (DAKO, Carpinteria, CA, USA) was used to develop the horseradish peroxidase (HRP)-streptavidin complex.

The assessment of CD31-positive capillaries was performed on 10 cross sections for each heart by two independent observers who were unaware of the experimental group from which the heart samples were derived. Each observer counted the number of CD-31-immunostained capillaries on 5 focal fields (photographed at × 400 magnification) for each slide and the means of values were considered as the data. For the evaluation of capillary proliferation, we measured only the immunostaining of CD31 in the capillaries that were identified thanks to vessel diameter (< 10 μm) and to the absence of outer layers besides endothelial.

### Statistical analysis

Data are reported as mean ± standard deviation. Analysis of variance (ANOVA) and Bonferroni’s correction for *post hoc* comparisons were used to test significant differences within and between sedentary and trained groups at different time points. Correlations between MMP-2, MMP-9 and CD31-positive capillaries were examined with Pearson correlation coefficient (*r*). Values were considered significantly different at P ≤ 0.05.

## Results

### Analysis of CD31 expression as a marker of capillary growth

**Figure 1:**
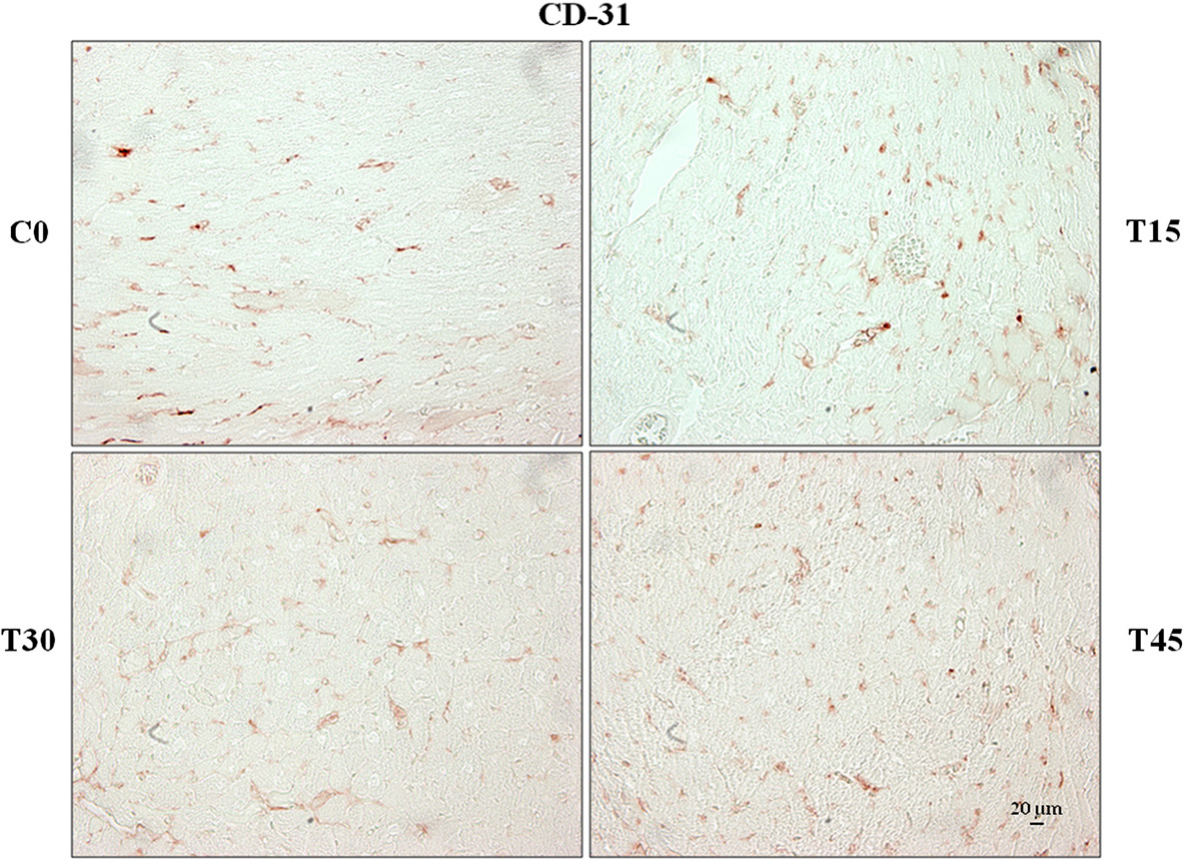
**Capillary proliferation in myocardium following aerobic training.** The number of CD31-posive capillaries was evaluated with immunostaining analyses in the left ventricle sections from trained (T15; T30 and T45) and control (C0) mouse hearts.

**Figure 2:**
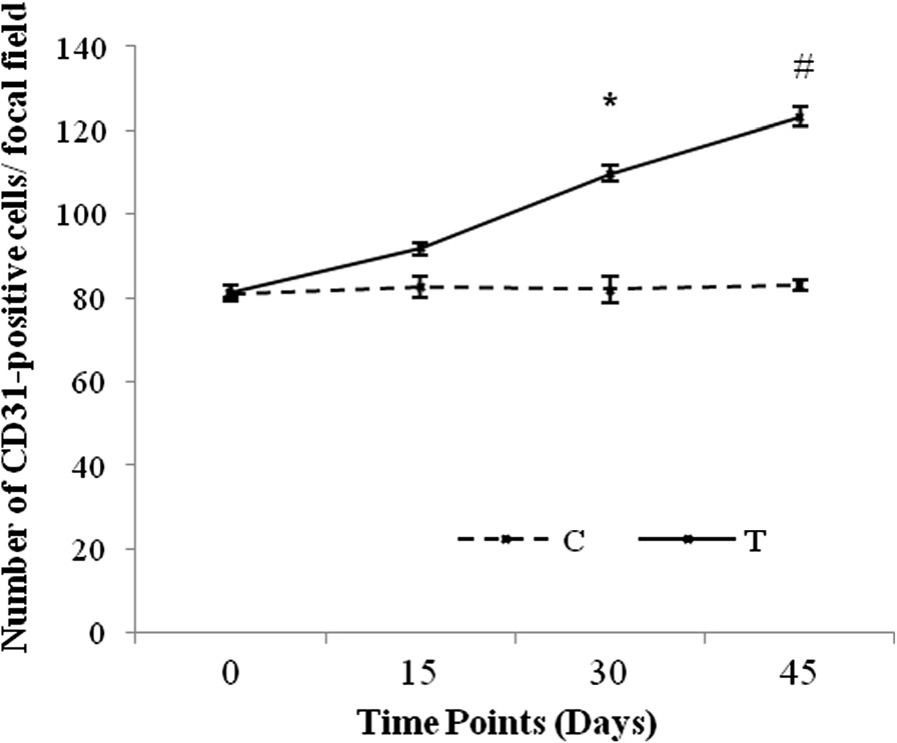
**Assessment of CD31-positive capillaries.** Two observers counted the number of CD31-immunostained capillaries on 10 cross sections for each heart and the means of values were considered as the data. Bonferroni’s multiple comparison test was used to analyze the significant differences within and between sedentary (C) and trained (T) groups at different time points. *P < 0.05 T30 and T45 vs. C samples and T15; #P < 0.05 T45 vs. T30).

It is known that CD-31, also called PECAM-1, is a glycoprotein expressed specifically on the surface of endothelial cells [18]. As shown in the Figure 1, CD31 immuno-reactivity was exclusively localized in the vessels of control and trained mouse hearts. Immuno-staining analysis performed on the left ventricle from the control groups did not show any remarkable difference in CD31 expression and for this reason only C0 group was shown in the Figure 1. The number of CD31-positive capillaries progressively increased in response to endurance training; however, only groups T30 and T45 showed a significant increase compared to corresponding control animals (T30: 109.75 ± 1.71 vs. C30: 82.00 ± 3.16 and T45: 123.25 ± 2.36 vs. C45: 83.00 ± 1.41; p < 0.01; Figure 2). Moreover, CD31 expression was significantly higher in groups T30 and T45 than in T15 animals, and in T45 than T30 group (T30: 109.75 ± 1.71 and T45: 123.25 ± 2.36 vs. T15: 91.75 ± 1.50; T45 vs. T30, p < 0.05; Figure 2). These data agree with previous results obtained by morphometric analyses showing a significant increase in the area occupied by capillaries in the left ventricles from the same trained mice [2].

### Evaluation of MMP-2 and MMP-9 activity in angiogenic hearts after exercise training

**Figure 3:**
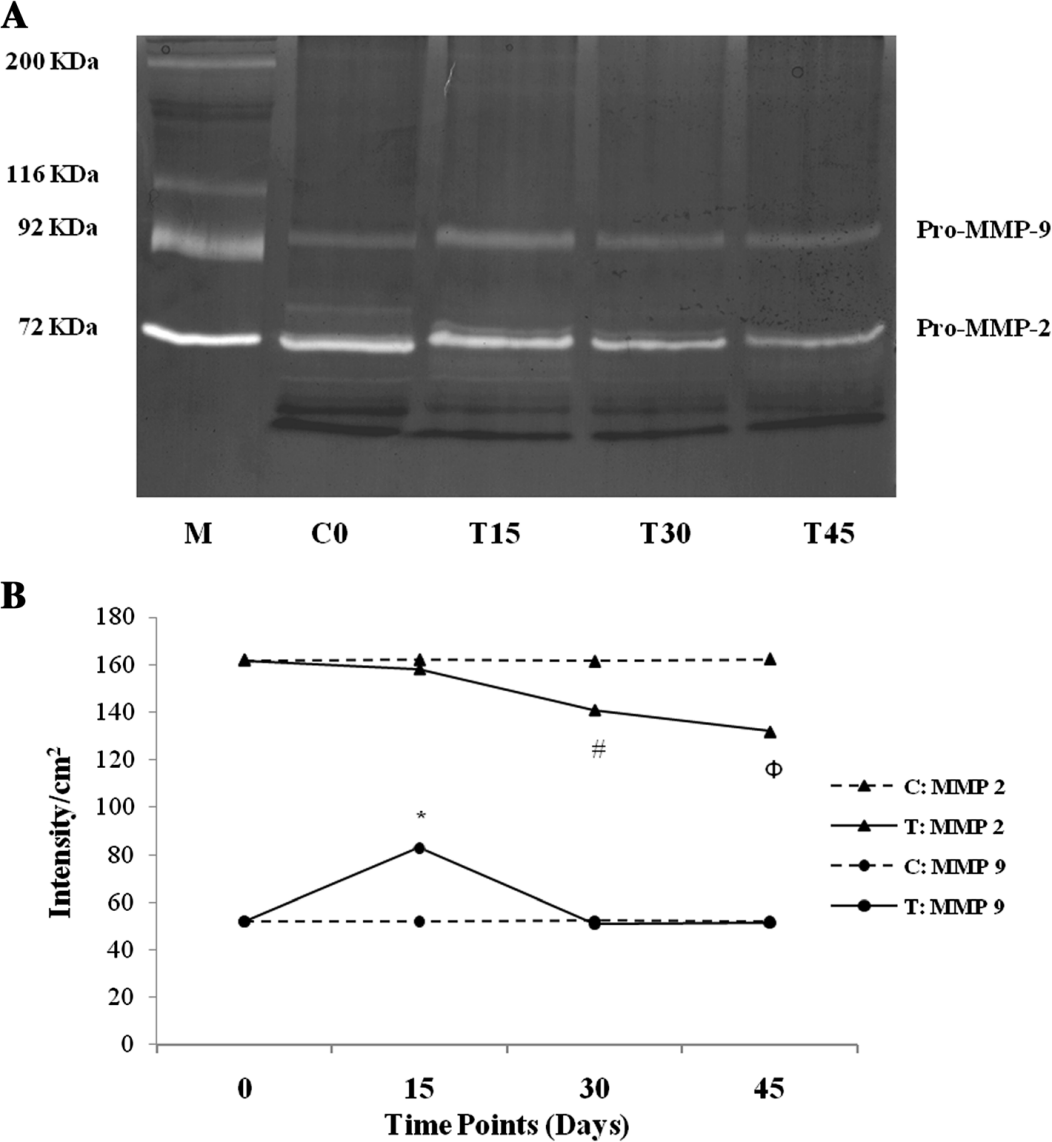
**Gelatinolytic activity of pro-MMP-2 and pro-MMP-9 in hearts from trained and control mice. (A)** Zymography analysis illustrates two digetion bands corresponding to the latent forms of MMP-2 and MMP-9. As control groups did not show any remarkable difference, only C0 group is shown. **(B)** Band intensity was quantified by computer-assisted image analysis calculating pixel number of signal per cm^2^ and significant differences within and between sedentary and trained groups were evaluated. (#P < 0.05 T30 and T45 vs. C samples and T15; ФP < 0.05 T45 vs. T30 *P < 0.05 T15 vs. C samples, T30, T45).

In the zymography analyses, we found only two digestion bands corresponding to the pro-enzymatic forms of MMP-2 and MMP-9 (Figure 3A). In agreement with Talhouk et al., the exposure of MMP-2 and MMP-9 pro-enzymes from our tissue extracts to SDS during gel separation procedure led to their activation without proteolytic cleavage [19].

In contrast to C samples, where the activity of pro-MMP-2 and pro-MMP-9 did not change during the time of study, a different pattern between pro-MMP-2 and pro-MMP-9 was noticed according to the time points of training protocol in trained mouse hearts (Figure 3A).

We indeed noticed that gelatinolytic activity of pro-MMP-9 has significantly increased in T15 animals compared with C (0, 15, 30 and 45), T30 and T45 groups (T15: 83.05 ± 1.32 vs. C0: 52.04 ± 1.16, T30: 50.96 ± 1.55 and T45: 51.58 ± 0.57; p < 0.05). By contrast, no difference was found between T30 and T45 animals (Figure 3B). Moreover, the levels of pro-MMP-9 of T30 and T45 were similar to C30 and C45 groups (Figure 3B).

With regard to the activity of pro-MMP-2, this gradually diminished in trained mouse hearts compared with those of control groups (Figure 3A). In detail, it was lower in groups T30 and T45 than T15, and C groups (T30: 140.87 ± 0.93, T45: 131.85 ± 1.350, vs. T15: 158.00 ± 0.80 and C0: 161.90 ± 0.86; p < 0.05) and in T45 than T30 mice (T45: 131.85 ± 1.35 vs. T30: 140.87 ± 0.93 p < 0.05). T15 and C groups did not show any significant difference (Figure 3B). Comparing the gelatinolytic activity of pro-MMP-2 and pro-MMP-9, we observed that pro-MMP-2 levels were higher than pro-MMP-9 either in sedentary and trained mouse hearts (Figure 3B). Further, pro-MMP-9 levels were not correlated with the number of CD31-positive cells (*r* = −0.37; p = 0.63); whereas pro-MMP-2 showed a strong and inverse correlation with capillary proliferation (*r* = −0.99; p = 0.0076).

### Analysis of MMP-2 and MMP-9 localization in response to endurance training

**Figure 4:**
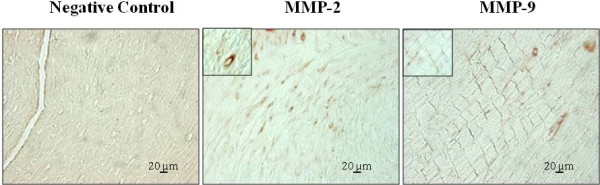
**Localization of MMP-2 and MMP-9 in the left ventricle sections from T15 hearts.** Nonimmune mouse serum was used as a negative control (NC).

Figure 4 shows that MMP-9 staining was mainly localized in myocardiocytes and less evident in capillaries. Conversely, MMP-2 immuno-reactivity was more intense in capillaries and slightly in myocardiocytes (Figure 4). No difference has been observed in the localization of these enzymes between trained and control groups.

## Discussion

This study has demonstrated that the activity of pro-MMP-2 and pro-MMP-9 is differently modulated during cardiac angiogenesis induced by aerobic training. Most previous studies have reported data concerning MMPs activity after a single acute bout of exercise either in the circulation [20,21] and in muscle tissue [14,22]. In the present investigation, we have examined the adaptive responses of MMP-2 and MMP-9 activities after 15, 30 and 45 days of low-moderate intensity endurance program. In the zymography analyses, we found the latent form of both MMPs either in control and trained groups and the absence of their activated form. This might be due to a quick decline in the half-life of these enzymes after they were activated in the extracellular matrix as also described for MMP-9 in mouse skeletal muscle by Demestre et al*.*[23]. However, the activity of MMP-2 was detected in rat cardiac muscle after 72, 96 and 120 hours of acute swimming training with an anaerobic component [24]. Therefore, the modality and intensity of exercise might affect the maturation and stability of these MMPs.

In detail, our results have shown that the amount of pro-MMP-9 was much lower than that of pro-MMP-2 either in control and trained groups and this is consistent with the dissimilar regulation of these enzymes at transcriptional level. Indeed, it is known that the basal synthesis of MMP-9 is absent or very little and can be induced by a wide variety of cellular signals through the activation of a proximal activator protein-1 (AP-1) site [25]. Conversely, MMP-2 gene is constituently expressed in most tissues and its promoter does not contain proximal AP-1 site, but distal enhancer elements modestly induced by cytokines and growth factors [26]. Moreover, we have found an unlike pattern between pro-MMP-2 and pro-MMP-9 according to the time and intensity of aerobic training program. The highest peak of pro-MMP-9 activity occurred after 15 days in which the exercise training was performed at the lowest intensity of the endurance protocol. In the mice’ hearts, trained for 30 and 45 days, we did not notice any pro-MMP-9 variation compared with control groups. On the contrary, there was no change in pro-MMP-2 after 15 days of training compared with control group, but this enzyme gradually decreased below the baseline level after 30 and 45 days of training. Similar results have been observed after 70 minutes of a 3% uphill running exercise in tendon-related connective tissue [27]. In this study, pro-MMP-2 decreased immediately and tended to further decrease one days post exercise. In contrast, pro-MMP-9 increased immediately post exercise and remained elevated up to three days after exercise.

Further studies carried out in the skeletal muscle have argued that both these MMPs are regulated not only by different stimuli according to the training protocols but also through mechanisms specific of cell type or tissue [14]. For instance, in rat cardiac cells, MMP-2 promoter is subjected to a novel form of transcriptional regulation compared with the other tissues. Here, the involvement of AP-1 site in the transcriptional regulation of MMP-2 is associated with cardiac cellular response to damaging stress, such as ischemia and infarction leading to the ventricular remodelling process [26]. Therefore, different expression and time patterns of MMP-2 and MMP-9 induced by exercise in cardiac and skeletal muscle might be explained with their involvement in dissimilar cellular process.

In our study, MMPs appear to be engaged in the exercise-related angiogenesis of hypertrophic myocardium. The gelatinolytic activity of pro-MMP-9 was not correlated to capillary proliferation trend (CD-31 expression pattern) and its up-regulation before the capillary increase suggests an involvement of this enzyme in the initial steps of angiogenesis such as also postulated by other studies [21,28]. Conversely, pro-MMP-2 levels were inversely correlated to capillary proliferation induced by aerobic training. The decrease in the latent form of this enzyme might be explained with an exercise-induced increase in the cleavage of pro-MMP-2 to produce its active form that is released in order to carry out its angiogenic functions. Indeed, it has been reported a coordinated expression of MMP-2 and MT1-MMP, known to activate pro-MMP-2, in the growth of new capillaries induced by chronic skeletal muscle stimulation [10,14].

Another regulation mechanism of MMPs is due to the activity of specific inhibitors called tissue inhibitors of metalloproteinase (TIMPs). TIMP-2 is known to bind most effectively to MMP-2, whereas TIMP-1 has a high affinity to MMP-9 [29]. A study carried out in human tendon has shown that the activity of these inhibitors was affected by exercise and associated to the pro-MMP-2 and pro-MMP-9 levels [27].

A dissimilar sensitivity by pro-MMP-2 and pro-MMP-9 to angiogenic-related mechanical stimuli induced by exercise such as stretching, shear stress, chronic electrical stimulation, hypoxia or vibration might underlie their different pattern as indicated by several studies [10,30].

In the present study, the participation of MMP-2 to angiogenic process is also supported by its localization prevalent in capillaries suggesting these as the primary sites of MMP-2 production. In contrast, the widespread presence of pro-MMP-9 in myocardiocytes indicates these cells as the main source of this enzyme and a different role with respect to MMP-2 in cardiac angiogenesis. Selective spatiotemporal regulation of pro-MMP-2 and pro-MMP-9 has been also reported in trained skeletal muscle [14] and during myocardial remodelling after heart infarction [17].

## Conclusions

Our results add significant information on the state of the art about the mechanisms of cardiac vascular growth in response to aerobic exercise. It is evident that pro-MMP-9 and pro-MMP-2 activity is regulated according to the time and intensity of endurance training protocol and associated with the capillary growth in hypertrophic mouse hearts. Pro-MMP-9 activity increases after 15 days of low-intensity exercise when neo-capillarisation is still not significant; whereas pro-MMP-2 activity gradually decreases in a training dose-dependent manner and it is inversely correlated with capillary proliferation. Further studies are certainly needed to understand the role of these MMPs in heart angiogenesis and metabolic responses.

Developing knowledge about MMPs involvement in cardiac angiogenesis regulation through exercise can contribute into drawing specific training protocols for improving the performance of healthy and diseased human hearts.

## Competing interests

The authors declare that they have no competing interests.

## Authors’ contributions

MB was the main researcher and was responsible for study design, interpretation of data and draft of manuscript; GB was responsible for study design, acquisition of data and draft of manuscript; AB was responsible for the statistical analysis, FF conceived the study and participated in its design; AP conceived the study and participated in its design, AP helped to draft the manuscript. All authors read and approved the final manuscript.

## Funding

This study was supported by laboratory research funds at University of Palermo.
